# Nutrition, lifestyle, and cognitive performance in esport athletes

**DOI:** 10.3389/fnut.2023.1120303

**Published:** 2023-05-18

**Authors:** Jenna B. Goulart, Logan S. Aitken, Saman Siddiqui, Marisa Cuevas, Jacqueline Cardenas, Karen M. Beathard, Steven E. Riechman

**Affiliations:** ^1^Department of Kinesiology and Sport Management, Texas A&M University, College Station, TX, United States; ^2^Department of Nutrition, Texas A&M University, College Station, TX, United States

**Keywords:** esports, cognitive performance, protein, micronutrients, athletes, cognition, dairy

## Abstract

**Introduction:**

Electronic sports, termed esports, is a growing athletic activity in which high levels of attention and cognitive performance are required. With its increasing popularity and competitiveness, interest in strategies to improve performance have emerged. Improving esports athlete performance, namely cognitive endurance, and resilience, may lie in nutritional or lifestyle factors. The Nutrition, Vision, and Cognition in Sport Studies (IONSport) investigated nutritional and behavioral factors that can influence cognition via 3-dimensional multiple objects tracking test (3DMOT) via Neurotracker X (NTx) software. The purpose of this study was to characterize the lifestyle of high level esports athletes with detailed nutrition, sleep, and physical activity assessments, and their association to gaming related cognitive performance.

**Methods:**

103 male and 16 elite female esports athletes aged 16 to 35 years old completed surveys, food records, and cognitive testing sessions over 10 days. Participants were instructed to maintain their normal dietary and lifestyle habits.

**Results:**

There were positive significant associations between average NTx scores and the following nutrients: magnesium, phosphorous, potassium, sodium, zinc, selenium, thiamin, niacin, vitamins B6 and B12, folate, cholesterol, saturated, polyunsaturated, and monounsaturated fats, omega-6 and omega-3 fatty acids, and choline. Majority of participants did not meet recommended dietary allowances (RDAs) for these micronutrients nor the recommended intakes for dairy, fruit, and vegetables. There was a significant (*p* = 0.003) positive (*r* = 0.272) association between total vegetable intake and average NTx score. There was a significant negative association (*p* = 0.015) with our final sustain session, which measured cognitive resilience, and the Stanford Sleepiness Scale score. Repeated measures analysis was done with these groups over the 18 core NTx sessions. There were significant (*p* = 0.018) differences between the two groups with those who consumed the recommended amount of protein or more performing significantly better on NTx over the 18 sessions than those that did not consume enough protein. Those who consumed the recommended intakes for riboflavin, phosphorous, vitamin B12, and selenium performed significantly better over the 18 core NTx sessions than those that did not meet the recommended amounts.

**Discussion:**

The need for a nutrition intervention that is rich in protein, vitamins, and minerals is warranted in this population.

## 1. Introduction

Electronic sports, or esports, are athletic activities consisting of various avenues of digital gameplay including video and personal computer (PC) modalities (1). The esports industry has been growing exponentially with an estimated global revenue of 66.6 million in 2021 (2) and an expected worldwide audience of 577.2 million by 2024 (3). Esports athletes undergo intense training leading up to competitions (4) with the potential to win prize purses of $121 million in 2017 and $100 million in the 2018 *Fortnite* competition season (5).

In many of the games, elevated levels of attention and cognitive performance are needed to remain competitive in the esports world. The available research on esports and cognitive performance has primarily been on action-based video games, showing that these esports athletes have a higher processing speed than the controls (6). However, a recent review showed that players of non-action-based video games did not have a significantly greater cognitive ability (7). Cognitive testing, for esports athletes, traditionally utilizes a battery of tests from the validated National Institutes of Health (NIH) Toolbox for Neurological and Behavioral Function. That is, these tests singly evaluate executive function, episodic memory, language, processing speed, and attention (8). Uniquely, task-switching tests, such as 3-Dimensional (3D) Multiple Object Tracking (MOT) integrate these skills (9) and may represent a more realistic, yet controlled testing environment (10).

With the growing esports industry and the popularity of avid gaming, keys to improving performance are crucial. As with traditional athletes, one aspect of improving performance is optimizing nutrition with a primary emphasis on neurological function rather than just skeletal muscle or cardiovascular systems. Esports athletes share many attributes with traditional athletes (11) and thus utilize similar training paradigms to those who compete in physical sports (12). Research on nutritional factors and esports athletes has been limited to individual micronutrients like creatine, or non-nutritive substances such as caffeine (13–16). Specifically, a recent study investigating ingestion of a caffeine metabolite, paraxanthine, was executed by Yoo et al. to determine its efficacy in improving cognitive performance. After supplement ingestion, cognitive performance increased in some but not all cognitive tests. Regardless, caffeine and its metabolites have been vastly studied for their performance benefits and have been found to promote an overall increase in cognitive outcomes with the consequences of diminishing returns, caffeine addiction and continued elevation of doses to achieve benefits (17).

A descriptive study that analyzed esports athletes of different ranks in Germany found that most of the athletes assessed engaged in positive health behaviors as defined by a health score, adequate physical activity, and body mass index (BMI). However, none of the groups, as defined by their ranking, consumed the recommended five servings of fruits and vegetables assessed by a single question of intake, although the professional esports athletes consumed more fruits and vegetables than any other group (18).

Nutrition is just one aspect of overall lifestyle that could benefit esports performance. Other components of lifestyle to achieve optimal performance include physical activity and sleep; however, they have not been studied extensively in high level esports athletes. A global study investigating health behaviors and esports athletes found that the majority of those sampled did not meet the World Health Organization (WHO) guidelines (19) of 150–300 min moderate physical activity per week or 75–150 min of intense/vigorous activity per week for physical activity despite maintaining a normal BMI. They also found that most esports athletes did not smoke and consumed less alcohol than the global average. Interestingly, players in the top 10% of their gaming rank reported greater amounts of physical activity than 90% of the total esports athletes sampled (20). Esports athletes are generalized as having poor sleep due to their increased time spent gaming. This colloquialism has some truth, as a study investigating three professional esports teams demonstrated that these athletes were sleeping less than 7 h per day and had significantly increased awake time after initial sleep onset with subsequent daytime sleepiness. It is hypothesized that these professional athletes have training times that could impact their sleep opportunity (21). Everyday esports athletes could also experience the deleterious effects of lack of sleep as evidenced by Ketelhut et al. (22); however, as Peracchia and Curcio explain, there is conflicting evidence to support the impact of sleep based on results from the literature (23).

While these previous findings in esports athletes are beneficial, the importance of nutrition, physical activity, and sleep on performance in high level esports athletes is lacking. Therefore, this study aimed to observe the role of nutrition, physical activity, and sleep on cognitive training/testing. It was hypothesized that esports athletes would not be consuming a well-balanced diet, have adequate sleep habits, or meet the recommended levels of physical activity, and these factors would be associated with impairment of cognitive performance.

## 2. Methods and materials

### 2.1. Participants

**Figure 1:**
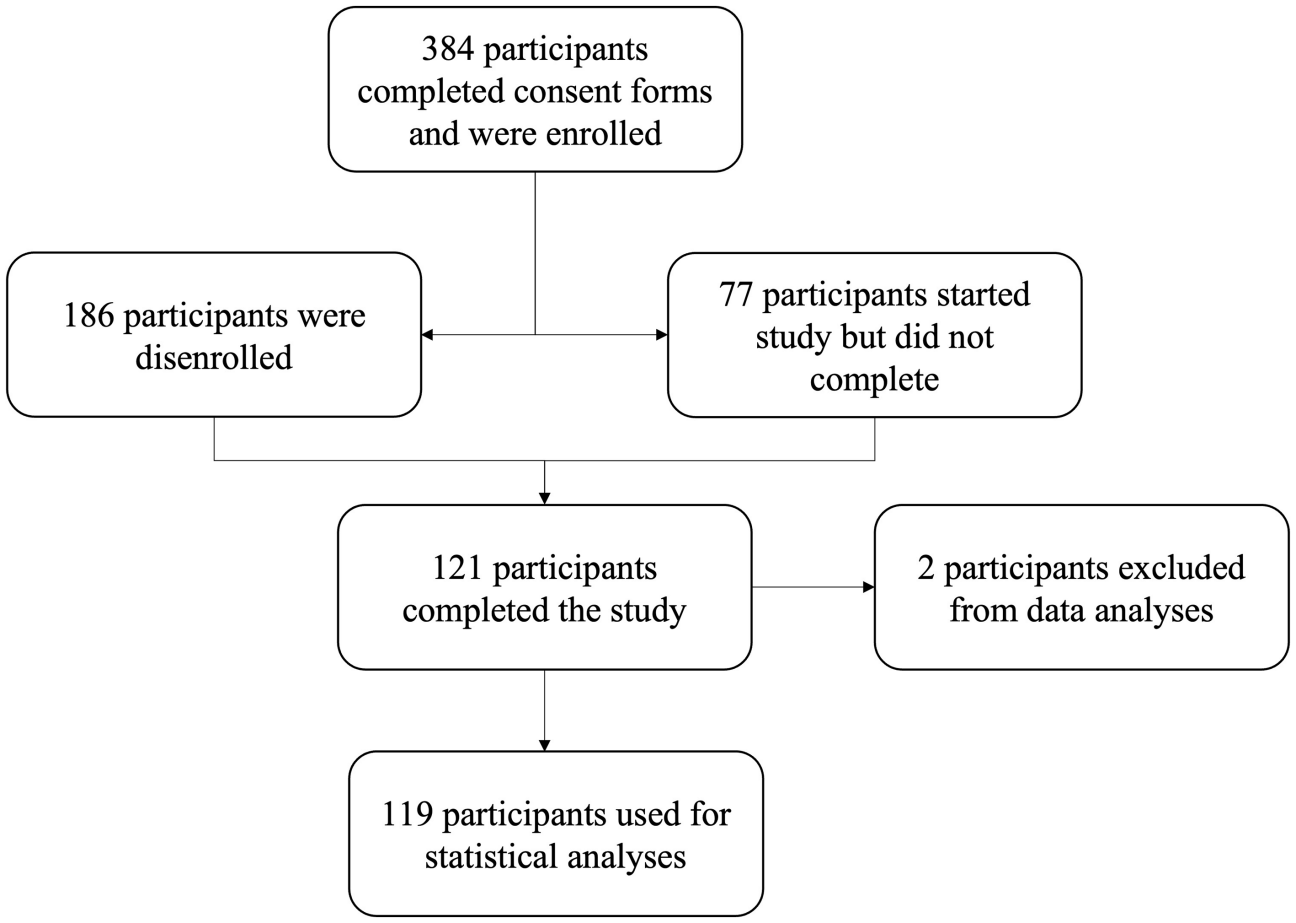
Subject enrollment data.

Healthy males (*n* = 103) and females (*n* = 16) volunteered to participate in this fully remote study. All participants were classified as professional, elite, or avid esports athletes. Participants were included in this study if they were between the ages of 16 to 35 years old with acceptable vision (best-corrected vision of 20/40 or better in each eye). Participants were excluded if they had a pacemaker, had an untreated psychiatric disorder, were color blind, had a diagnosis or condition of vertigo, macular degeneration, diabetic retinopathy, glaucoma, retinitis pigmentosa, optic neuropathy, retinal vascular occlusions, strabismus, or other autoimmune disorders related to visual health. Participants completed an informed consent form before the preliminary questionnaires ensuring their eligibility for the study. All methods and study procedures were approved by the Texas A&M University Institutional Review Board for Human Subjects in research. A total of 384 participants consented; however, 186 withdrew. Additionally, 77 participants started the study but did not finish. One hundred twenty-one participants completed the study; however, two were not included in data analyses due to incomplete data (Figure 1).

### 2.2. Recruitment

Participants were recruited using social media platforms including Instagram, Twitter, Facebook, and Discord, and through emailing esports teams. Additionally, flyers were distributed in College Station, and Austin, Texas. Participants were prompted to fill out a Google form, which provided emails for further communication. Consent, and assent forms, if needed, were signed. Participants were then assigned a participant ID followed by a brief informational Zoom meeting to answer questions and provide precise instructions on conducting the study.

### 2.3. Surveys

The following forms were completed via REDCap (an online survey and study management platform): a Gaming History Questionnaire, Demographics, Medical History, Vision Screening, Pittsburgh Sleep Quality Index (PSQI), Modifiable Activity Questionnaire and a daily pretesting survey. The Gaming Questionnaire, a survey developed by our lab team, was designed to assess participant skill levels and gaming habits. Questions included tournament rankings, gamer rank, and games played. The Demographics, Medical History, and Vision Screening surveys were used to obtain baseline health data and study eligibility. The PSQI, a questionnaire that generates a score based on individual and bed partner feedback, characterized sleep behavior and clarified sleep patterns and quality. A higher PQSI score reflected worse sleep quality (24). The Modifiable Activity Questionnaire was used to assess the frequency and duration of several types of physical activity for the past year and lifetime (25).

### 2.4. Nutrition tracking and monitoring

Participants were asked to continue their normal eating behaviors and physical activity throughout the study. They were instructed to complete 10 days of food records via the Automated Self-Administered 24-Hour Dietary Recall (ASA24) software, a standardized and validated tool from the National Cancer Institute (26), which allows for accurate estimation of energy and nutrient intake. In ASA24, participants were guided through a series of questions as they completed their food records using multiple passes and probes to enhance memory and recall of foods consumed and provided visual aids to determine portion sizes. At study completion, nutrition reports were created and sent to participants after being reviewed by a Registered Dietitian, and if necessary, feedback was provided.

### 2.5. Physical activity and sleep monitoring

The LETSCOM Smart Band was used as a wearable device that monitored steps, heart rate (HR), and sleep (LETSCOM. (2017) Smart Band ID107Plus HR). Participants were instructed to consistently wear the Smart Band on their nondominant wrist, only removing it for necessary events (e.g., showering). It was worn for at least 8 continuous days after an initial full charge. Data from the tracker was obtained from the VeryFit Pro app, which is the associated app for the tracker. Participants entered this data into their pretesting survey, which they completed prior to every cognitive training session.

### 2.6. Visual cognitive performance testing

During each of the 8 days of cognitive training, participants entered the following information on a pretesting survey: recent physical activity, fluid intake, most recent urine color via a validated urine color scale (27), HR, readiness to perform, body composition, Stanford Sleepiness Scale (28), and hours of sleep the previous night.

Participants completed 20 cognitive training sessions, remotely, using the Neurotracker X (NTx) 3D software program over 8 training days. Days one and eight included the baseline testing consisting of four training sessions (three core training sessions and one sustain training session) while days two through seven consisted of two core training sessions. Participants were instructed to set up their screen according to the NTx guidelines and confirm that the distance between the eyes and the display was the same as the width of the display. For example, participants using a 17-inch laptop should have their eyes approximately 17-inches away from the screen. In addition, the center of the screen remained at eye level during training. Participants conducted the testing in a dimly lit room with no distractions while wearing 3D glasses. Participants, who relied on glasses for corrected vision wore them under the 3D glasses when training.

NTx trained four aspects of perceptual-cognitive function: division of attention while tracking multiple objects, large visual field, performance at one’s maximal speed threshold, and 3D visual cues. Each core training session required tracking the spatial location of four pre-identified target spheres initially highlighted from the other four. Once identified, these spheres became identical in color to the four other spheres. All eight identical spheres moved among each other at a given speed within a 3D virtual space: passing in front of or behind each other, colliding with each other or the edges of the screen, or changing directions. After six-seconds (6 s) of movement, the spheres stopped, and the participant identified the four pre-identified spheres. If the subject selected all four of the correct spheres, the speed of sphere movement increased for the next 6 s-trial. If one or more spheres were missed, the speed of sphere movement decreased for the next trial in a staircase pattern. Subjects performed 20 trials within a single training session obtaining a “speed threshold,” which was the level at which the participant correctly tracked and selected the correct objects 50% of the time. The final speed threshold for each training session and the progression over 20 sessions were the primary outcomes of cognitive performance.

The sustain training sessions, used to measure cognitive resilience, included one 60 s trial at 8 s duration. Four targets would highlight and then return to the starting color. The first trial was self-paced with all targets continuously moving. At the conclusion of the session, the participant received a speed threshold score. Sustain sessions trained and assessed the participants’ ability to maintain fluid attention over time and were used as a stamina assessment, which is potentially related to the ability to stay focused during game play.

### 2.7. Statistical analyses

Descriptive statistics were performed on all data. Initial student’s t-test was performed to assess baseline differences between males and females. Correlations were performed using Spearman’s rho to guide further investigation. Analyses with significant correlation (*p* < 0.05) were considered key variables and used for repeated measures ANOVA. For repeated measures, variables were recoded into new variables to generate groups for the analyses. The significance level was p < 0.05 for all analyses. All analyses were performed using IBM SPSS Statistics 29.

## 3. Results

### 3.1. Subjects

Three hundred eighty-four participants enrolled in the study; however, there are complete data sets for analyses on 119 participants (103 males and 16 females). A student’s t-test was performed, and there were no significant differences (*p* < 0.05) between male and female baseline characteristics (Table 1).

**Table 1:** Participant characteristics.

	Male				Female				*p* value
	*N*	Mean	Std. Dev	Min-Max	*N*	Mean	Std. Dev	Min-Max	
Age (yrs)	103	23.1	5.0	16–36	16	24.4	5.0	18–33	0.319
Height (in)	103	69.2	4.0	60.0–81.0	16	68.8	3.1	63.0–74.0	0.724
Weight (lbs)	103	187.6	59.9	97.0–420.0	16	176.5	52.6	120–300.0	0.477
BMI	103	27.5	8.4	16.0–63.9	16	26.0	6.7	18.6–39.50	0.241

^*^p < 0.05. Student’s test performed assessing significant differences between male and female baseline characteristics. No significant differences.

**Figure 2:**
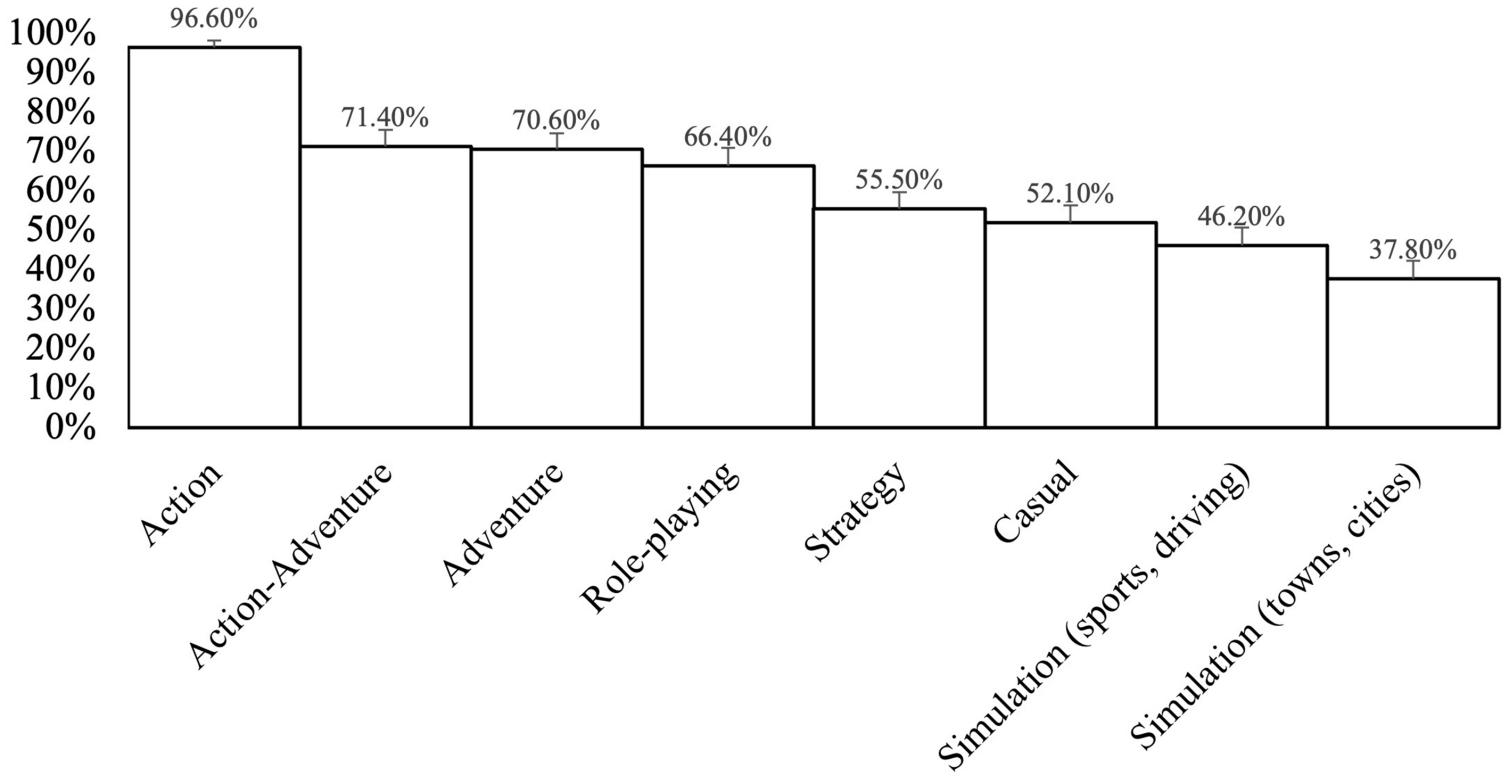
Game types played by participants. Error bars represent SEM for column percent.

As shown in Table 2, participants gamed an average of 6.33 days with 4.82 h spent gaming in one sitting (Table 2). The most popular game type, with 96.6% of total participants playing, included those in the category of action (platformers, shooters, racing, and fighting) and action-adventure, with 71.4% of total participants playing (action games with strong storylines) (Figure 2). An example of the action game type is *Fortnite* and an example of the action-adventure game type *is Halo.*

**Table 2:** Gaming frequency of participants.

	*N*	Mean	Std. Dev	Min–Max
Days spent gaming per week	119	6.3	1.1	2–7
Hours spent gaming in one sitting	119	4.8	2.0	3–14

### 3.2. Nutrition

Participants were instructed to maintain their normal eating habits for the study. They recorded all food and beverage intake for 10 days via ASA24. However, 4 participants included in analyses did not fully adhere to the protocol and only recorded their food and beverage intake for 1 to 9 days (n = 4). The average macronutrient intake of participants is shown in Table 3. The average intake over the 10 days was 1,852 kilocalories (kcals) with 79.9 grams (g.) protein, 76.2 g. fat, and 210.8 g. carbohydrates (Table 3). Moisture represented water intake and was measured in milliliters (ml.). Participants consumed 2,041.8 ml. of total water (including moisture from foods, beverages, and separate water intake).

**Table 3:** Macronutrient intake over 10 days.

	*N*	Mean	Std. Dev	Min–Max
Kilocalories	119	1852	698	521–4,136
Protein (g)	119	79.9	32.9	21.6–203.6
Total Fat (g)	119	76.2	30.8	22.1–193.8
Carbohydrates (g)	119	210.8	91.1	36.0–592.4
Moisture (mL)	119	2042	907	179–4,862

Spearman’s correlations were performed using average NTx score (speed threshold) and all micronutrient data to determine variables of interest for further investigation. Micronutrients of interest, as shown in Table 4, were determined from significant (*p* < 0.05) correlations between average NTx score and the nutrient and *a priori* analysis based on our prior work. There were significant positive associations between average NTx scores and the following nutrients: magnesium, phosphorous, potassium, sodium, zinc, selenium, thiamin, niacin, vitamins B6 and B12, folate, cholesterol, saturated, polyunsaturated, and monounsaturated fat, omega-6 and omega-3 fatty acids, and choline (Table 4). Average intakes over the 10-day period and recommended intakes were analyzed with most participants not meeting USDA guidelines for magnesium, zinc, folate, omega-6 and omega-3 fatty acids, vitamin D, and choline. Most participants exceeded cholesterol, sodium, and saturated fat recommendations (Table 4).

**Table 4:** Micronutrient intake over 10 days.

	*N*	Mean	Std. Dev	Min–Max	% Participants not meeting recommendation	Spearman’s rho	*p* value
Magnesium	119	239.0	96.2	59.1–626.5	95.7%	0.166	0.070
Phosphorous	119	1250.2	463.2	331.7–2937.9	11.2%	0.195	0.033
Potassium	119	1992.0	773.8	676.0–4882.9	NA	0.211	0.021
Sodium	119	3352.8	1193.8	1185.4–8341.2	16.5%	0.218	0.017
Zinc	119	10.2	4.4	2.4–26.7	69.0%	0.232	0.011
Selenium	119	113.3	46.8	33.1–278.1	4.3%	0.254	0.005
Thiamin	119	1.5	0.6	0.3–3.6	36.4%	0.203	0.026
Niacin	119	26.9	13.6	4.7–76.8	21.6%	0.224	0.014
Vit. B6	119	2.3	1.7	0.4–9.3	27.3%	0.204	0.026
Folate	119	329.3	145.0	87.2–866.5	76.9%	0.191	0.037
Vit. B12	119	5.2	3.5	0.7–20.3	13.8%	0.181	0.048
Cholesterol	119	291.5	168.6	57.4–1003.0	63.6%	0.213	0.020
Saturated fat	119	25.6	11.5	6.8–65.4	*66.1%	0.173	0.059
Monounsaturated fat	119	26.3	10.8	7.8–62.7	NA	0.252	0.006
Polyunsaturated fat	119	17.3	7.1	4.4–46.2	NA	0.277	0.002
Omega-6	119	15.3	6.4	3.8–40.9	57.0%	0.275	0.002
Omega-3	119	1.6	0.7	0.4–4.5	52.1%	0.227	0.013
Vit. D	119	3.9	4.0	0.4–25.2	97.4%	0.157	0.088
Choline	119	285.6	136.3	54.5–843.9	94.2%	0.245	0.007

^*^The recommendation for saturated fat is less than 10% of total kcal intake. Percent of participants not meeting recommendations is based on average participant caloric intake. ^*^NA indicates there is no current USDA recommendation for this nutrient. Recommendations are based on USDA Dietary Guidelines for Americans 2020–2025. Spearman’s rho is association to average Neurotracker scores.

**Figure 3:**
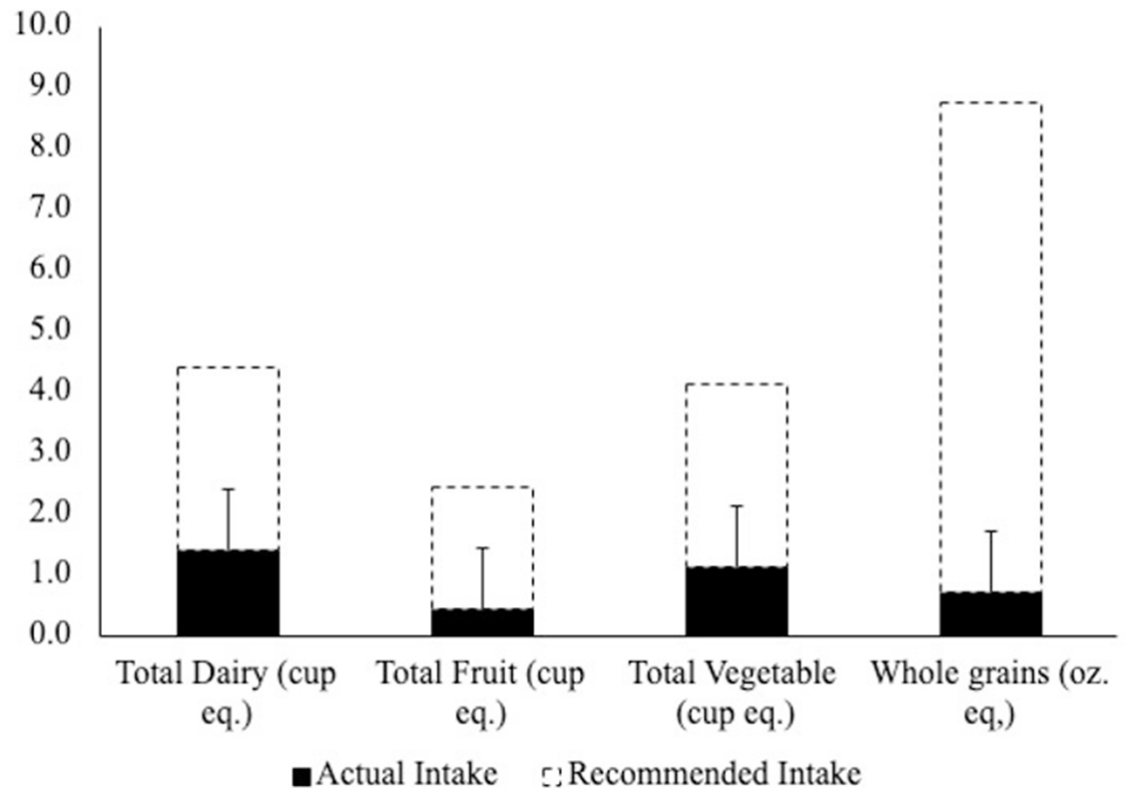
Dietary guidelines for americans 2020–2025 Food Group Intakes vs. Recommendations. Error bars represent + SD from mean.

Spearman’s correlations between these dietary factors and average NTx scores were performed and used to guide further analysis (Table 5). There was a significant (*p* = 0.003) positive (*r* = 0.272) association between total vegetable intake and average NTx score. In comparison to the Dietary Guidelines for Americans 2020–2025 participants did not meet recommendations for total vegetables, fruits, whole grains, or dairy intakes (Figure 3).

**Table 5:** Food groups, fiber, caffeine, and added sugars.

	*N*	Mean	Std. Dev	Min-Max	Recommendation	Spearman’s Rho	*p* value
Total Dairy (cup eq.)	119	1.4	0.8	0.1–4.7	3	0.065	0.483
Total Fruit (cup eq.)	119	0.4	0.6	0.0–3.4	2	0.065	0.478
Total Vegetable (cup eq.)	119	1.1	0.6	0.2–3.5	3	0.272	0.003
Whole grains (oz. eq,)	119	0.7	0.9	0.0–3.7	8	−0.048	0.603
Fiber (g)	119	13.1	5.6	3.6–37.1	14 g/ 1,000 kcal	0.177	0.054
Caffeine (mg)	119	74.6	90.7	0.0–520.3	NA	−0.001	0.990
Added Sugar (g)	119	14.1	10.8	0.3–54.0	NA	0.166	0.070

### 3.3. Physical activity and sleep

Participants wore an activity tracker (LETSCOM Smart Band) for each day they participated in cognitive testing resulting in 8 consecutive days of wearing the device. Average daily steps for participants were 3,941 steps with 2.64 total miles traveled per day walking (Table 6). The average resting HR was 68.6 beats per minute (bpm) with an average minimum HR of 57.5 bpm and an average maximum HR of 95.7 bpm (Table 6). Participants slept for an average of 445.2 min or 7.42 h. The average time awake after sleep onset was 9.85 min (Table 7). Sleep health was also measured by two subjective sleep questionnaires, the PSQI and Stanford Sleepiness Scale, and our pretesting survey. The average PSQI global score was 6.13. A PSQI global score greater than 5 is associated with severe or moderate sleep disturbances (24). The Stanford Sleepiness Scale measured participant alertness via a self-assessed seven-point scale with 1 being very alert and 7 being excessively sleepy. The participant was also able to select X, which indicated they were barely conscious due to sleepiness. For our data analysis, X was calculated as an 8 (28). The average Stanford Sleepiness Scale score was 2.77.

**Table 6:** Physical Activity as measured by a wearable device.

	*N*	Mean	Std. Dev	Min-Max
Avg distance (miles)	114	2.6	4.5	0.1–35.4
Avg steps	114	3,941	2,817	336–21,173
Avg calories	113	4740.1	9223.4	15.2–9223.4
Avg heart rate (bpm)	114	68.6	13.9	44–146
Max heart rate (bpm)	114	95.7	36.8	53–193
Min heart rate (bpm)	114	57.5	9.6	3–100

**Table 7:** Sleep health as measured by a wearable device.

	*N*	Mean	Std. Dev	Min-Max
PSQI global score	119	6.1	2.7	1.0–14.0
Stanford sleepiness scale	114	2.8	1.0	1.0–6.0
Avg sleep minutes	112	445.2	76.0	225.0–682.0
Avg awake minutes	112	9.9	3.2	1.0–13.0

### 3.4. Visual cognitive performance testing

For 8 consecutive days, participants completed visual cognitive performance testing via NTx. On the first (day 1–baseline) and last (day 8–elevated baseline) day of the testing, participants completed three core sessions and one sustain session. During the middle days of the study (days 2 through 7), participants completed two core sessions. All sessions were completed in one sitting. Speed threshold, the level at which the participant correctly tracked and selected the correct objects 50% of the time, was determined for each session. The average speed threshold was 1.52. Additionally, a sustain score was given for each sustain trial completed. On average, the initial sustain score was 1.04 while the average final sustain score was 1.53 (Table 8). The sustain score was the speed threshold score derived from the sustain trial. Additionally, NTx reference data, which highlights the difference in performance between non-athletes, elite athletes, and professional athletes was considered in the evaluation and showed that our participants performed with the elite group but could not reach the same level of performance as professional athletes (29).

**Table 8:** Neurotracker X Scores.

	*N*	Mean	Std. Dev	Min–Max
Average NTx Score	119	1.52	0.43	0.34–2.75
Low NTx Score	119	0.83	0.39	0.01–2.20
Top NTx Score	119	2.18	0.58	0.73–4.71
Sustain 1 Score	118	1.04	0.44	0.12–2.45
Sustain 2 Score	115	1.53	0.53	0.19–3.41

### 3.5. Sleep and visual cognitive resilience

There were no significant associations between NTx speed threshold scores and thus, visual cognitive performance, and sleep health. However, there was a significant negative association (*r* = −0.230, *p* = 0.015) between the final sustain session and the Stanford Sleepiness Scale score. The sustain sessions measure cognitive resilience, and it was found that participants with an increased Stanford Sleepiness Scale score performed worse on the sustain session. Specifically, the significant negative association was in the second sustain session, although there was a downward trend in the first sustain session (*r* = −0.069). Associations of average sleep hours, as measured by a wearable device, and cognitive performance were not significant but also showed a negative association.

### 3.6. Physical activity and cognitive performance and resilience

There were no significant associations between physical activity metrics and cognitive performance or cognitive resilience.

### 3.7. Nutrient impact on cognitive performance

Protein was normalized for body weight for each participant in grams protein per kilogram of bodyweight (g/kg). Dichotomous groups were created with one group representing individuals who consumed less than 0.8 g/kg and the other group being those who consumed 0.8 g/kg or more. This protein value was used based on the USDA Dietary Guidelines for Americans 2020–2025 protein recommendations (30). Repeated measures analysis was done with these groups over the 18 core NTx sessions. There were significant (*p* = 0.018, Observed Power *β* = 0.812) differences between the two groups with those who consumed the recommended amount of protein performing significantly better on NTx over the 18 sessions than those that did not consume enough protein (Figure Error! Reference source not found.).

Additionally, similar methods in transforming variables into above and below the recommended intakes were used for vitamin D, riboflavin, calcium, phosphorous, vitamins B6 and B12, selenium, zinc, niacin, and magnesium. Those who consumed the recommended intakes for riboflavin, phosphorous, vitamin B12, and selenium all performed significantly better over the 18 core NTx sessions than those that did not meet the recommended amounts. However, this result reflects analysis of uneven groups. Nonetheless, our data shows that most participants were not consuming these nutrients adequately and this was associated to reduced performance on NTx (Table 4).

## 4. Discussion

In this study, we examined the role of nutrition, physical activity, and sleep on cognition related gaming performance in esports athletes. We tested the assumption that most esports athletes do not consume a nutritious, well-balanced diet. We hypothesized that cognition related gaming performance, as shown through NTx cognitive testing scores, would be enhanced if esports athletes consumed proper nutrition, were physically active, and had quality sleep habits.

The results demonstrated that the average food intake of esports athletes did not meet the standards for a nutritious, well-balanced diet based on the USDA Dietary Guidelines for Americans 2020–2025 (30). The recommendation for total caloric intake is 2,400 kcals and 2000 kcals, for males and females, respectively, for this age group (30). However, the average intake of the 10 days of food and beverage logs was 1852kcals, which was below the recommendation for both males and females. The average macronutrient intake for participants included 79.9 g. protein, 76.2 g. fat, and 210.8 g. carbohydrates, which exceeds the RDA of 46 g. protein for females, 56 g. protein for males, and 130 g. carbohydrates for both males and females based on their age. However, on average, protein and carbohydrate intake were within acceptable macronutrient distribution ranges (AMDR) at 17.3 and 45.5%, while fat was slightly above the recommendation at 37% of total intake (30).

The majority of micronutrient intake did not meet the USDA Dietary Guidelines for Americans 2020–2025 (30). As shown in Table 4, the average intake of magnesium, zinc, folate, omega-6 and omega-3 fatty acids, vitamin D, and choline across the 10 days did not meet recommendations. However, there were positive significant (*p* < 0.05) associations between average NTx score and the following nutrients: magnesium, phosphorus, potassium, sodium, zinc, selenium, thiamin, niacin, vitamins B6 and B12, folate, cholesterol, saturated, polyunsaturated, and monounsaturated fats, omega-6 and omega-3 fatty acids, and choline. Micronutrients including thiamine, niacin, vitamins B6 and B12, folate, magnesium, and zinc were most closely associated with cognitive functioning and have been shown to influence cognitive performance through neurotransmitter synthesis, neuronal membrane and receptor modification, and energy metabolism (31). While key nutrients for improving performance were decreased, nutrients that may have an impact on health were increased and exceeded recommendations including average cholesterol, saturated fat, and sodium. A study analyzing cognitive performance in children found that saturated fat intake was related to longer reaction times during tasks that require more cognitive flexibility. In addition, high saturated fat and cholesterol intake were associated with higher switch costs demonstrating a decrease in working memory and reaction time (32). These negative effects on cognitive performance due to saturated fat and cholesterol could have impacted the esports athletes NTx scores, as NTx core sessions test an individual’s division of attention, reaction time, and memory.

The overall lack of micronutrient intake can be attributed to esports athletes not satisfying the Dietary Guidelines for Americans 2020–2025 for consumption of dairy, fruit, vegetables, and whole grains (30). The average recommended intake of dairy, fruit, vegetables, and whole grains, according to the Dietary Guidelines for Americans is 3.0 cups eq, 2.0 cups equivalents (eq.), 3.0 cups eq., and 8.0 oz. eq. respectively (30). As shown in Table 5, average total dairy, fruit, vegetables, and whole grains intake were, respectively, 0.4 cups eq., 1.1 cups eq., 0.7 oz. eq., and 1.4 cups eq., which are all much lower than the daily recommendations. This finding is consistent with a prior study that investigated esports athletes where they also did not meet the recommended servings of fruits or vegetables (18). However, as seen in Table 4, there is as significant (*p* = 0.003), positive (r = 0.272) association between total vegetable intake and average NTx score indicating that more nutritious foods improve cognitive performance. Prior research has found caffeine doses between 32 to 300 mg enhance various facets of cognitive performance including attention, vigilance, and reaction times (33–35). However, our results, as shown in Table 5, did not find a significant association between caffeine intake and cognitive performance. This could be due to the average caffeine intake being on the lower end at only 74.6 mg, which is about the equivalent of one cup of coffee. The esports athletes who habitually consume this lower dose of caffeine may not achieve performance benefits. The decrease in caffeine-induced benefits at the ergogenic level has been seen in athletes who are habitual caffeine users and, increasing caffeine doses has been required to maintain ergogenic effects (36), which may translate to maintaining cognitive performance enhancements as well.

Sedentary behavior is often linked to increased screen-time activities. Esports athletes have been assumed to have more of a sedentary lifestyle due to their increased hours spent on gaming. As seen in Table 2, the average days spent gaming per week was 6.3 days with 4.8 h spent gaming per day. When examining physical activity, which was measured through the participant’s wearable device, the average amount of steps the esports athletes took per day was 3,941 steps, and an average distance of 2.64 miles per day. This is much lower than the Centers for Disease Control’s (CDC) recommendation of 10,000 steps per day, the equivalent of about 5 miles (37). In addition, Table 6 shows that the average maximum HR was only 95.7 bpm, indicating that most esports athletes were not participating in moderate to vigorous intensity physical activity per day as the target HR for moderate-intensity activity is between 64–76% of max HR (37). This would have been 126–150 bpm for the male esports athletes and 125–149 bpm for the female esports athletes based on their average age. These data suggest that the esports athletes were physically inactive, which may have negatively affected their NTx scores. Chang et al. found a dose–response relationship between resistance exercise and cognitive performance where high-intensity exercise improves the speed of processing and moderate intensity exercise improves executive function (38). Another study evaluating the effects of exercise on gaming performance of *League of Legends*, a popular video game, found that 15 min of high -intensity interval training exercise prior to playing the video game enhanced performance as the video gamers improved their accuracy and capacity to eliminate targets (39).

Similarly, the increased hours esports athletes spend on gaming have been assumed to negatively impact their sleep quality. Esports athletes have been associated with having non-traditional sleep characteristics and, in fact, have been found to have shorter overall sleep durations of about 7 h, including a later sleep onset and sleep offset than traditional athletes (40). This is consistent with the current study as the participants slept for an average of 7.42 h (Table 7). In addition, the average global PSQI score, which was used to examine overall sleep quality, was 6.1, indicating poor sleep, as a PSQI global score > 5 is associated with severe or moderate sleep disturbances (24). Furthermore, as shown in Figure 5, there was a negative association (*p* = 0.015) with the final sustain session and the Stanford Sleepiness scale (28). As the score increased on the Stanford Sleepiness Scale, the participants’ sustained session score decreased. Sleep restriction was shown to impair various aspects of cognitive performance including working memory and attention-based tasks such as sustained attention, visuo-spatial attention, serial attention, reaction time, and subtraction tasks (41). These executive functions are crucial to being a successful esports athlete as they need to have quick reaction times and sustained attention, so improving sleep health will be instrumental in enhancing esports performance.

It was expected that esports athletes would have higher NTx scores than the average individual due to the cognitive skills associated with being a successful esports athlete. However, as displayed in Table 8, the NTx score, which was the speed threshold, was 1.52. The speed threshold refers to the participant correctly tracking and selecting the object 50% of the time. When compared to NTx standards, as shown in Figure 6, the esports athletes’ average score was not vastly different from elite-amateurs and was lower than professionals. This finding coincides with a study investigating the cognitive profile of experienced video game players where it was found that they had higher processing speeds, task switching abilities, and responded faster on a Stroop test, displaying a quick reaction time, but made significantly more errors compared to individuals with little or no video game experience. This could be due to esports athletes employing the use of pre-firing, a strategy used in action games, where they react to a stimulus prior to its occurrence, which may lead to an increased error rate (6).

**Figure 4:**
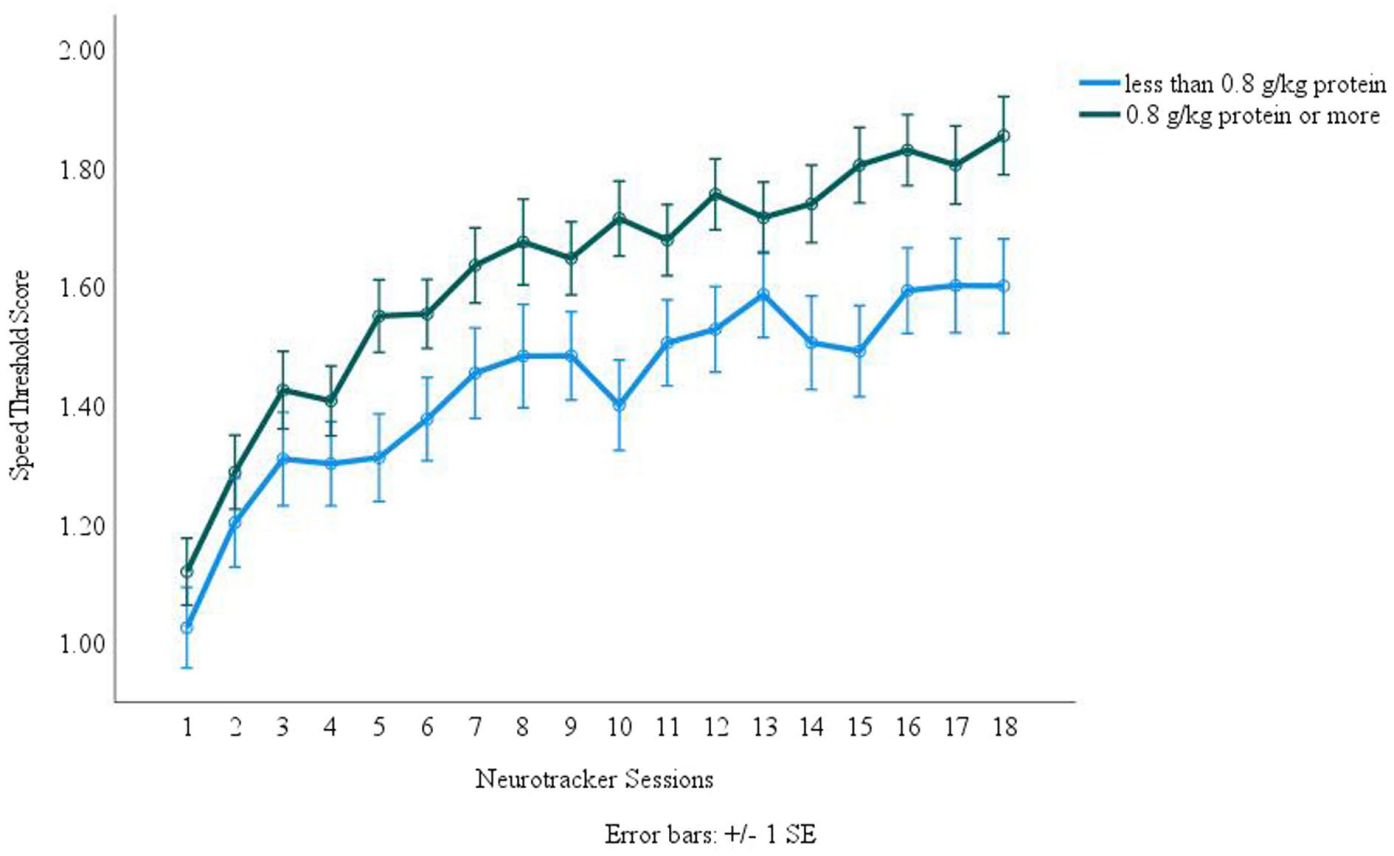
Protein Intake and Neurotracker Performance. Error bars represent =/− 1 SEM.

Specific macro and micronutrients and their relationship to cognition related gaming performance were also analyzed. The results demonstrated, as seen in Figure 4, that participants who consumed 0.8 g/kg protein or more performed better during the 18 NTx core sessions than those who consumed less protein. This could in part be due to protein intake enhancing working memory and episodic memory, especially when task demands are increased (42). In addition, daily tyrosine intake has been positively associated with fluid intelligence and working memory (43). A study investigating macronutrient composition and risk of mild cognitive impairment found that individuals who had a high percent protein intake were at reduced risk for mild cognitive impairment (44). Similarly, a review on protein intake and cognitive performance by van de Rest., van der Zwaluw, & de Groot demonstrated that there is an established link between protein intake and cognition (45). Furthermore, improvements to cognitive performance and function come from the amino acids found in proteins which support the genesis and turnover of neurotransmitters (46).

It was also observed that participants who consumed the recommended intakes of riboflavin, phosphorous, vitamin B12, and selenium all performed better than those who did not meet the recommended intakes. Prior studies have also found similar positive effects between riboflavin, vitamin B12, and selenium and cognitive function; however, this research has mainly been done on an elderly or diseased population. For example, riboflavin has been shown to improve multi-dimensional cognitive functioning in middle-aged and elderly people (47) and vitamin B12 has been found to positively affect memory functioning scores that were tested using the Wechsler Memory Scale-Revised in mild cognitive impairment patients (48). Previous research has also linked vitamin B12 deficiency to possible cognitive decline and cognitive function although higher quality evidence is needed (49). Additionally, in a study investigating older Chinese adults, high selenium intake was linked to having a high global cognition score and better memory, possibly due selenium’s antioxidative properties (50). On the other hand, high phosphorus intake has typically been associated with impaired cognitive functioning. One study using middle-aged participants found that an increased phosphorus intake was associated with a lower composite cognitive score (51). Similarly, another study investigating United States (US) veterans found that increased dietary phosphorus intake led to increased serum phosphorus levels, which was associated with a greater risk of incident dementia (52).

**Figure 5:**
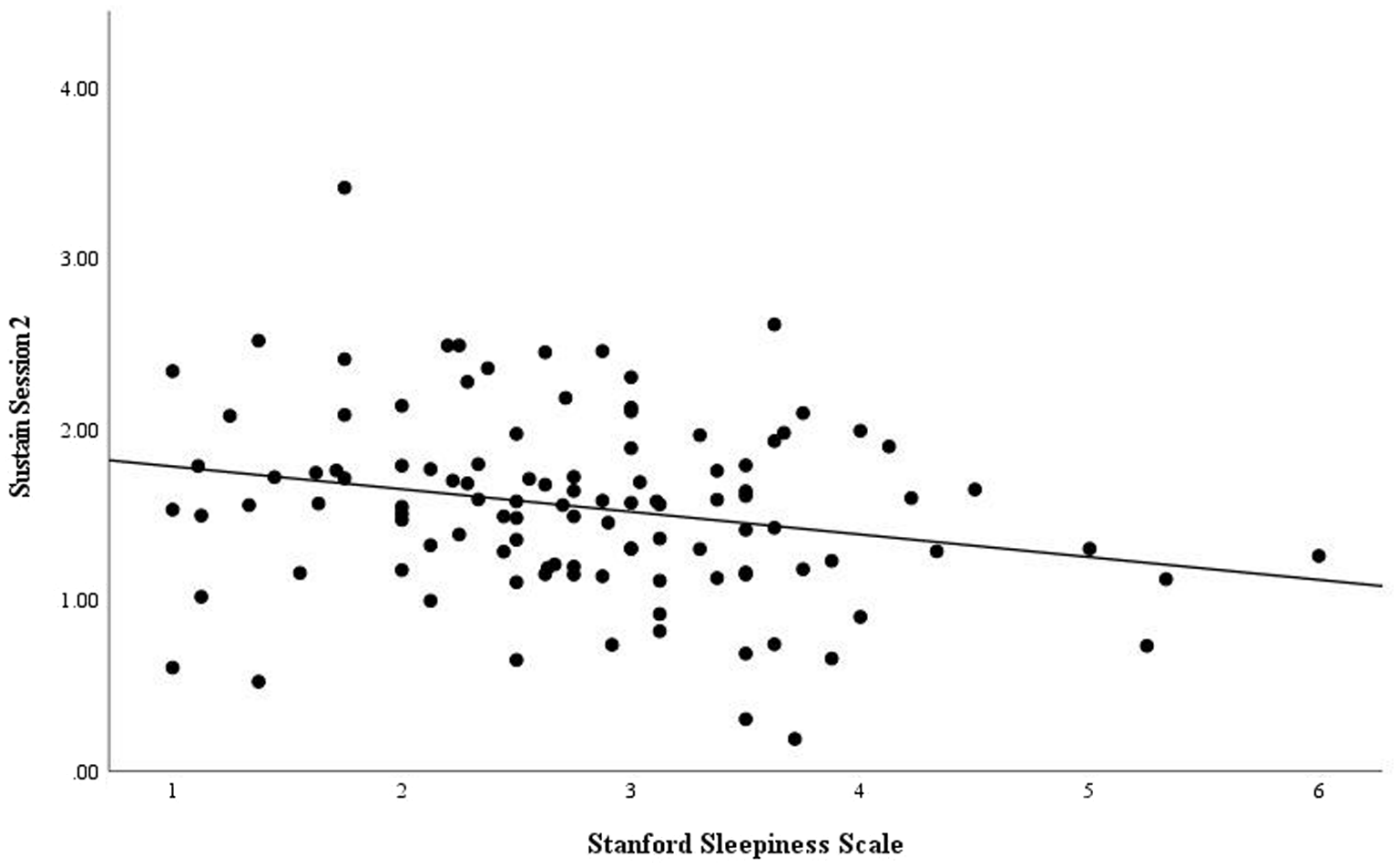
Association of cognitive resilience and Stanford Sleepiness Scale.

Our study was limited in the use of a 100% remote platform, so we could not assess true compliance to study protocols. In addition, participants may not have been entirely truthful or accurate when completing the surveys/questionnaires. However, this study provided us the opportunity to capture sleep, physical, dietary habits, and cognitive performance in a group of elite esports athletes from across the US that would have otherwise been inaccessible. Another limitation was the use of food records as it is known that this method results in underreporting of intake. Despite this, ASA24 is a clinically validated tool developed from the NIH that uses images for portion sizes and a second pass technique for interviewing thus mitigating underreporting. NTx core session scores were increased with higher protein intake as seen in Figure 5. NTx scores were expected to increase from consumption of adequate nutrients, but as seen in Table 4, most participants did not meet recommended intakes, which could have played a role in the scores being lower than expected. Additionally, sleep quality as measured by the Stanford Sleepiness Scale, had an impact on cognitive resilience with those with worsened quality being associated with worsened performance. Furthermore, esports athletes could improve their performance through lifestyle modifications.

**Figure 6:**
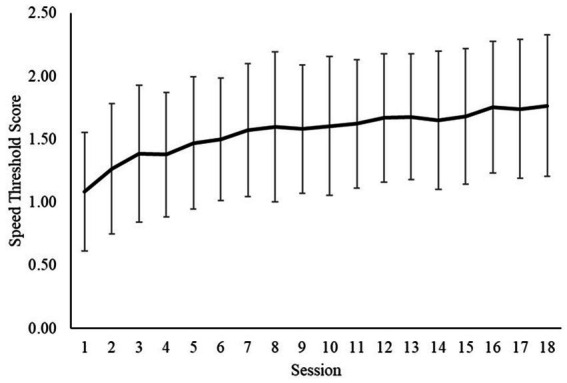
Average neurotracker Score over 18 core sessions. Error bars represent +/− SD from mean.

Our results indicate that sufficient protein and certain micronutrients are associated with improved cognitive performance in esports athletes. In addition, physical activity levels and sleep habits are not optimized in this population which if corrected may help to further optimize cognitive performance. It remains to be determined how a nutrition intervention that is high in protein and rich in vitamins and minerals could attenuate cognitive fatigue rather than solely improve performance. For example, dairy has a promising potential for improving cognitive performance in esports athletes due to its balanced and varied food matrix (53). In particular, bovine milk is rich in vitamins A, D, B, and C and minerals including calcium, magnesium, and potassium (54) which could make it a promising nutritional intervention for esports athletes (55–57). Total dairy, protein, vegetable, fruit, and whole grain intake, all of which are components of a well-balanced diet were not sufficiently consumed in this population, to correct macro and micronutrients deficiencies that essential for optimizing cognitive functioning. However, much work is needed to identify specifics nutrients, foods and food matrices that can be used to optimize high level cognitive performance applicable to esports athletes as well as many other high-level performers.

## Data availability statement

The raw data supporting the conclusions of this article will be made available by the authors, without undue reservation.

## Ethics statement

The studies involving human participants were reviewed and approved by Texas A&M University Institutional Review Board for Human Subjects in research. Written informed consent to participate in this study was provided by the participants' legal guardian/next of kin.

## Author contributions

SR is responsible for study design and conception, data analysis, and revision of the manuscript. KB and SR supervised all data collection, analysis, and manuscript editing. JG executed study experiments, supervised study activities, analysed data, and wrote the draft of the manuscript. LA is responsible for study recruitment and the draft of the discussion along with final edits. JG, LA, SS, MC, and JC collected data. All authors contributed to the article and approved the submitted version.

## Funding

The authors declare that this study received funding from DairyMax Inc. The funder was not involved in the study design, collection, analysis, interpretation of data, the writing of this article, or the decision to submit it for publication.

## Conflict of interest

The authors declare that the research was conducted in the absence of any commercial or financial relationships that could be construed as a potential conflict of interest.

## Publisher’s note

All claims expressed in this article are solely those of the authors and do not necessarily represent those of their affiliated organizations, or those of the publisher, the editors and the reviewers. Any product that may be evaluated in this article, or claim that may be made by its manufacturer, is not guaranteed or endorsed by the publisher.
